# Prevalence and molecular characteristics of ESBL and AmpC β -lactamase producing *Enterobacteriaceae* strains isolated from UTIs in Egypt

**DOI:** 10.1186/s13756-020-00856-w

**Published:** 2020-12-10

**Authors:** Ebtisam S. Mohamed, Rasha M. M. Khairy, Soha S. Abdelrahim

**Affiliations:** 1 Department of Microbiology and Immunology, Faculty of Medicine, Minia University, Minia, 61511 Egypt; 2 Department of Biomedical Sciences, College of Medicine, King Faisal University, Al Hofuf, Saudi Arabia

**Keywords:** *Enterobacteriaceae*, AmpC β -lactamase, Extended-spectrum β-lactamase (ESBLs)

## Abstract

**Background:**

Infections caused by *Enterobacteriaceae* are mainly treated with the β-lactam antibiotics, nevertheless, the emergence of species with plasmid-borne β-lactamases has decreased the efficacy of these antibiotics. Therefore, continuing studies on the resistance pattern of different regions is important for assessment of proper antimicrobial therapy protocols. The study aimed to characterize extended-spectrum β-lactamase (ESBL) and AmpC β –lactamase (AmpC) producing *Enterobacteriaceae* isolated from community-acquired UTIs in Egypt.

**Methods:**

Out of 705 urine samples, 440 *Enterobacteriaceae* isolates were investigated to detect ESBL and AmpC β -lactamases producers by phenotypic and molecular methods.

**Results:**

Out of 440 *Enterobacteriaceae* isolates, 311 were identified as ESBL producers by phenotypic testing. ESBL genes were detected in 308 isolates. *BlaCTX-M-type* was the most prevalent 254 (81.6%), out of them *blaCTXM-15* was the commonest (152, 48.8%) followed by *blaCTX-M-1* (140, 45%), *blaCTX-M-8* (72, 23.1%) and lastly *blaCTX-M-2* (4, 1.3%).

*blaTEM* gene also was detected in a high rate (189, 60.7%). Two hundred and thirty-five (75.5%) of ESBL producers harbored *blaCTX-M* in combination with *blaTEM* and/or *blaSHV* genes. Multiple drug resistance in the ESBL-producers was significantly (*P* < 0.05) higher than in non–ESBL producers. Imipenem was the most effective drug against ESBL producers. Among 35 cefoxitin resistant isolates, 18 (51.4%) identified as carrying AmpC genes by multiplex PCR. Within AmpC β *-*lactamase genes, DHA gene was the predominant gene (15, 42.3%). CIT and MOX genes were also present, but in a low rate (5, 14.2% and 4, 11.4%) respectively. Co-existence of multiple AmpC genes was detected exclusively in *K. pneumoniae* isolates. *E. coli* isolates harbored DHA gene only*.* However, FOX gene was not detected in the study isolates. Seventeen of isolates carrying AmpC genes were also positive for ESBL genes.

**Conclusion:**

The study shows that the prevalence of ESBL producing *Enterobacteriaceae* spread in south Egypt is alarming, however AmpC β -lactamase production is not so high.

## Background

*Enterobacteriaceae* are the most common pathogens causing urinary tract infections (UTIs) [1]. Increasing rates of antimicrobial resistance among *Enterobacteriaceae* strains decrease the options for empiric treatment of these infections [2]. These pathogens are the main bacteria found to be associated with extended-spectrum β-lactamase (ESBL) production [2]. Infections caused by ESBL-producing strains are considered a serious global health concern [3, 4] as these infections are associated with higher morbidity and mortality rates [5]. ESBL production is a mechanism of resistance in which the beta-lactam ring of antimicrobials such as penicillins and cephalosporins is hydrolyzed [6]. Until 2000s, *blaSHV* and *blaTEM* types of ESBLs used to be the commonest ESBL genotypes found in *Enterobacteriaceae* strains [7]. The corresponding genes were often found on plasmids that facilitate their rapid spread between different bacterial species [8, 9]. After that, *blaCTX-M* types were recorded as the commonest genotypes among *Enterobacteriaceae* strains causing human infections worldwide (particularly *blaCTX-M-15*) [10]. There are other variants of β-lactamases such as AmpC *β* -lactamase, that can mediate resistance to several antibiotics as penicillins, cephamycins (e.g., cefoxitin and cefotetan), and oxyimino-cephalosporins [11]. Resistance to broad-spectrum β*-*lactams mediated by ESBLs and AmpC *β* -lactamase enzymes has posed a great health burden [12], particularly in developing countries where the resistance rates are high. Additionally, drug use guidelines and studies on this issue are not enough in these countries [13]. Due to a lack of solid data regarding the emergence of ESBLs and AmpC β -lactamase enzymes from Egypt, particularly south Egypt, this study aimed to determine the prevalence of ESBLs and AmpC β -lactamase production in *Enterobacteriaceae* isolated from patients suffering from community- acquired UTIs and characterize these strains using phenotypic and genotypic assays.

## Methods

### Study design

This prospective study was conducted in the Department of Medical Microbiology and Immunology, Faculty of Medicine, Minia University, Egypt from June 2018 to December 2018. Urine samples were obtained by simple random sampling method from patients with suggested community-acquired UTI in 3 teaching hospitals in Minia, Egypt; Minia university hospital, Suzan Mubarak University hospital and Renal university hospital. The study included 705 patients of both sexes and different ages attending the outpatient’s clinics or admitted to the inpatient’s wards (who developed symptoms within 48 h of admission), who had no history of antibiotics use in the last 2 weeks. Demographic and clinical history of the patients were recorded. The samples were collected using the clean-catch midstream urine sampling technique.

### Bacterial isolates

Calibrated 0.01 mL urine plastic loops were used to inoculate Urine samples on 5% blood agar and MacConkey agar plates. The plates were incubated for 24 h at 37 °C. Samples with suspected contamination and that had multiple organisms were excluded from the study. Urine samples with positive cultures with a colony count ≥ 10^5^ colony-forming units per milliliter (CFU/mL) were only included. Out of 705 non repetitive samples included in the study, 440 isolates of *Enterobacteriaceae* were identified. *Enterobacteriaceae* isolates were identified by the standard biochemical tests including IMViC (indole, methyl red, Voges-Proskauer, citrate utilization), sugar fermentation, urease, and motility tests. The identified isolates were confirmed by chromogenic media (CHROMagar™ Orientation, Paris, France) and kept in trypticase soy broth with sterilized 15% glycerol at − 20 °C for further examination. The sample size was calculated using the formula advanced by Kish, 1965 [14], Basing on results of a previous study on the prevalence of ESBL and AmpC β -lactamase production in Egypt by Wassef et al., 2014 [15].

### Antibiotic susceptibility testing

Disk diffusion method was used for identification of antibiotic susceptibility of the *Enterobacteriaceae* isolates to different antibiotics according to CLSI guidelines [16]. The used discs were; amoxicillin/clavulanic acid (AMC) 20 μg/10 μg, ceftazidime (CAZ) 30 μg, ceftriaxone (CRO) 30 μg, imipenem (IPM) 10 μg, amikacin (AK) 30 μg, gentamicin (CN)10 μg, nitrofurantoin (F) 300 μg, ciprofloxacin (CIP) 5 μg and cefoxitin (FOX) 30 μg (for detection of AmpC production) (Thermo Scientific™ Oxoid, UK). Resistance to three or more classes of antimicrobial agents is defined as Multiple drug resistance (MDR) [17].

### Screening for ESBLs -producing strains

According to the CLSI guidelines, isolates with inhibition zone size ≤22 mm with ceftazidime (CAZ) 30 μg and ≤ 25 mm with ceftriaxone (CRO) 30 μg were suggested to be ESBL-producers and subjected to further phenotypic and genotypic examination. Double-Disc Synergy Test (DDST) was used for confirmation of ESBL production. Standard (0.5 McFarland) inoculum of the study isolates were inoculated on Mueller Hinton agar plates. Ceftazidime (CAZ) (30 μg) and ceftriaxone (CRO) 30 μg discs were applied on agar 1.5 cm away from the center of amoxicillin-clavulanic acid (AMC) (20 μg/10 μg) disc and incubated at 35 °C for 18 h. Positive result is identified when the zone of inhibition is extended towards AMC (20 μg/10 μg) disc > 5 mm [18].

### Screening for AmpC β-lactamase-producing strains

Strains were screened using disk diffusion method in which cefoxitin (FOX) 30 μg disc was used. Isolates showing an inhibitory zone diameter ≤ 18 mm were suspected to be AmpC β-lactamase producers [19]. Disc Approximation Assay (D Test) was also performed; a blunting in the inhibitory zone (D shaped) around the CAZ (30 μg) towards the side of one of the inducers (IPM (10 μg), FOX (30 μg), and AMC (30 μg)) is considered as positive for inducible AmpC β-lactamase production [20].

### Molecular characterization of ESBLs and plasmid mediated AmpC β*-*lactamase genes

DNA extraction was done using QIAamp Mini kit (Qiagen, Hilden, Germany), according to the manufacturer’s instructions. All isolates that were phenotypically resistant to β-lactams were screened for ESBL genes by the polymerase chain reaction (PCR), Including *blaTEM, blaSHV*, *blaCTX-M* (1, 2, 8, 9, 15) genes. Presence of other resistance genes previously associated with plasmids encoding *blaCTX-M-15* as *aac(6′)-Ib-cr* was screened by PCR. A multiplex PCR was used to examine the presence of plasmid-mediated AmpC genes, including; MOX, CIT, DHA, and FOX genes. Amplified products were resolved on 2% agarose gel electrophoresis and visualized under a UV transilluminator (Biometra, Germany). The primer sequences and amplification conditions are shown in Table 1. Amplified products (one sample for each gene) sequences were analyzed (Applied Biosystems, USA), according to the BLAST software of the National Library of Medicine (http://www.ncbi.nlm.nih.gov/blast).

**Table 1:** PCR primers of the current study

Gene name	Primer sequence	fragment Size (bp)	Annealing Temperature	Reference
*blaTEM*	AAACGCTGGTGAAAGTA AGCGATCTGTCTAT	822	58	**[**21**]**
*blaSHV*	ATGCGTTATATTCGCCTGTG TGCTTTGTTATTCGGGCCAA	753	60	**[**21**]**
*blaCTX-M-1*	GGT TAA AAA ATC ACT GCG TC TTG GTG ACG ATT TTA GCC GC	850	55	[22]
*blaCTX-M - 9*	ATG GTG ACA AAG AGA GTG CA CCC TTC GGC GAT GAT TCT C	850	55	[22]
*blaCTX-M- 2*	F CGACGCTACCCCTGCTATT R CCAGCGTCAGATTTTTCAGG	552	52	[23]
*blaCTX-M − 8*	TCGCGTTAAGCGGATGATGC AACCCACGATGTGGGTAG	666	52	[23]
*blaCTX-M-15*	CACACGTGGAATTTAGGGACT GCCGTCTAAGGCCATAAACA	996	55	[24]
MOX	GCTGCTCAAGGAGCACAGGAT CAC ATT GAC ATA GGT GTG GTG C	520	64	[25]
FOX	AAC ATG GGG TAT CAG GGA GAT G CAA AGC GCG TAA CCG GAT TGG	190		
DHA	AAC TTT CAC AGG TGT GCT GGG T CCG TAC GCA TAC TGG CTT TGC	405		
CIT	TGG CCA GAA CTG ACA GGC AAA TTT CTC CTG AAC GTG GCT GGC	462		
*aac(6*_*)-Ib*	F: TTGCGATGCTCTATGAGTGGCTA R: CTCGAATGCCTGGCGTGTTT	482	55	[26]

### Statistical analysis

Statistical analysis of demographic, clinical and laboratory data of study subjects was performed using SPSS for windows version 19.0 (IBM, USA). The chi -square test was used for analyzing categorical variables. *P* value < 0.05 was considered statistically significant (two-tailed).

## Results

### Demographic data and distribution of *Enterobacteriaceae* strains

A total of 440 *Enterobacteriaceae* strains were isolated from urine specimens of 440 patients suffering from UTI. The mean age of the patients was 38.8 ± 12.5 years (range, 5–60 years). A total of 299 (68%) were females and 141 (32%) were males. The majority of isolates were *E. coli* (303/440 (68.9%), followed by *Klebsiella pneumoniae* (*K. pneumoniae)* (71/440, 16.1%), *Citrobacter* spp. (40/440, 9.1%), *Proteus* spp. (15/440, 3.4%) and *Enterobacter* spp. (11/440, 2.5%).

### Antimicrobial susceptibility and phenotypic identification

**Fig. 1:**
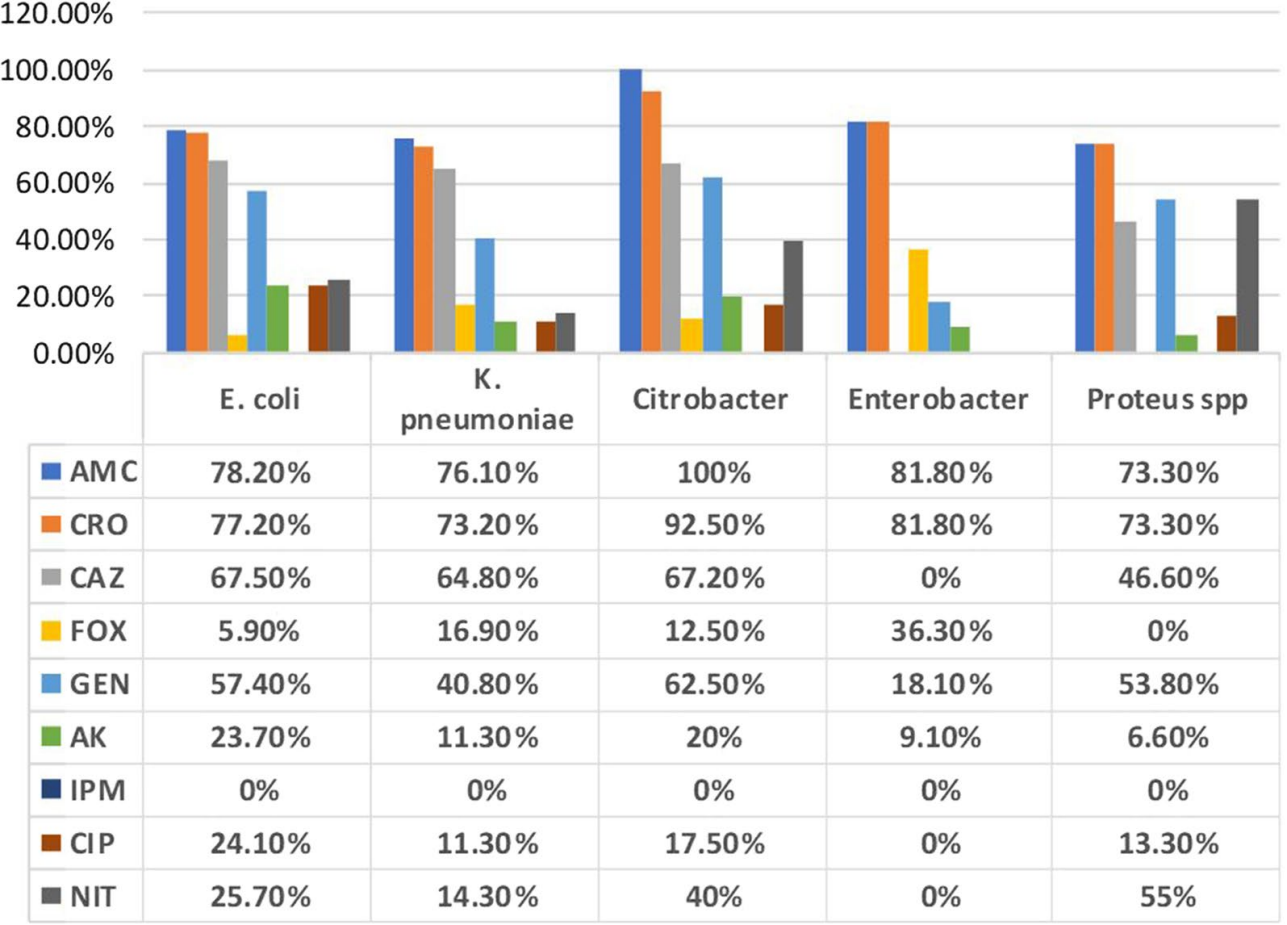
Antimicrobials resistance patterns of 440 *Enterobacteriaceae* isolates from UTIs. AMC; Amoxicillin Clavulanic acid, CRO, Ceftriaxone, CTZ; Ceftazidime, FOX; Cefoxitin, CN; Gentamicin, AK; Amikacin, IMP; Imipenem, CIP; Ciprofloxacin, F; Nitrofurantoin

Among 440 *Enterobacteriaceae* isolates tested for antimicrobial susceptibility, the resistance rates were; AMC (351/440, 79.7%), CRO (343/440, 77.9%), CAZ (289/440, 67.8%), GEN (238/440, 54.3), AK (90/440, 20.4%), CIP (90/440, 20. 4%), NIT (110/440, 25%), and FOX (35/440, 7.9%). All isolates were sensitive to IPM (Fig. 1). Antimicrobial susceptibility and phenotypic tests identified 311 (70.6%) isolates as ESBL producers and 35 (7.9%) isolates as AmpC β-lactamase producers (cefoxitin resistant). Induction test gave no positive results at all. Regarding distribution among different species; the frequency of ESBL production was 211/311 (69.6%) in *E. coli*, 53/71 (74.6%) in *K. pneumoniae,* 40/40 (100%) in *Citrobacter* spp. and 7/15 (46.6%) in *Proteus* spp. isolates. However, the frequency of suggested AmpC β-lactamase production (cefoxitin resistant) was 18/311(5.8%) in *E. coli,* 12/71 (16.9) in *K. pneumoniae,* and *5*/40 (12.5%) in *Citrobacter* spp. isolates.

### Genotypic characterization of ESBL producers

**Fig. 2:**
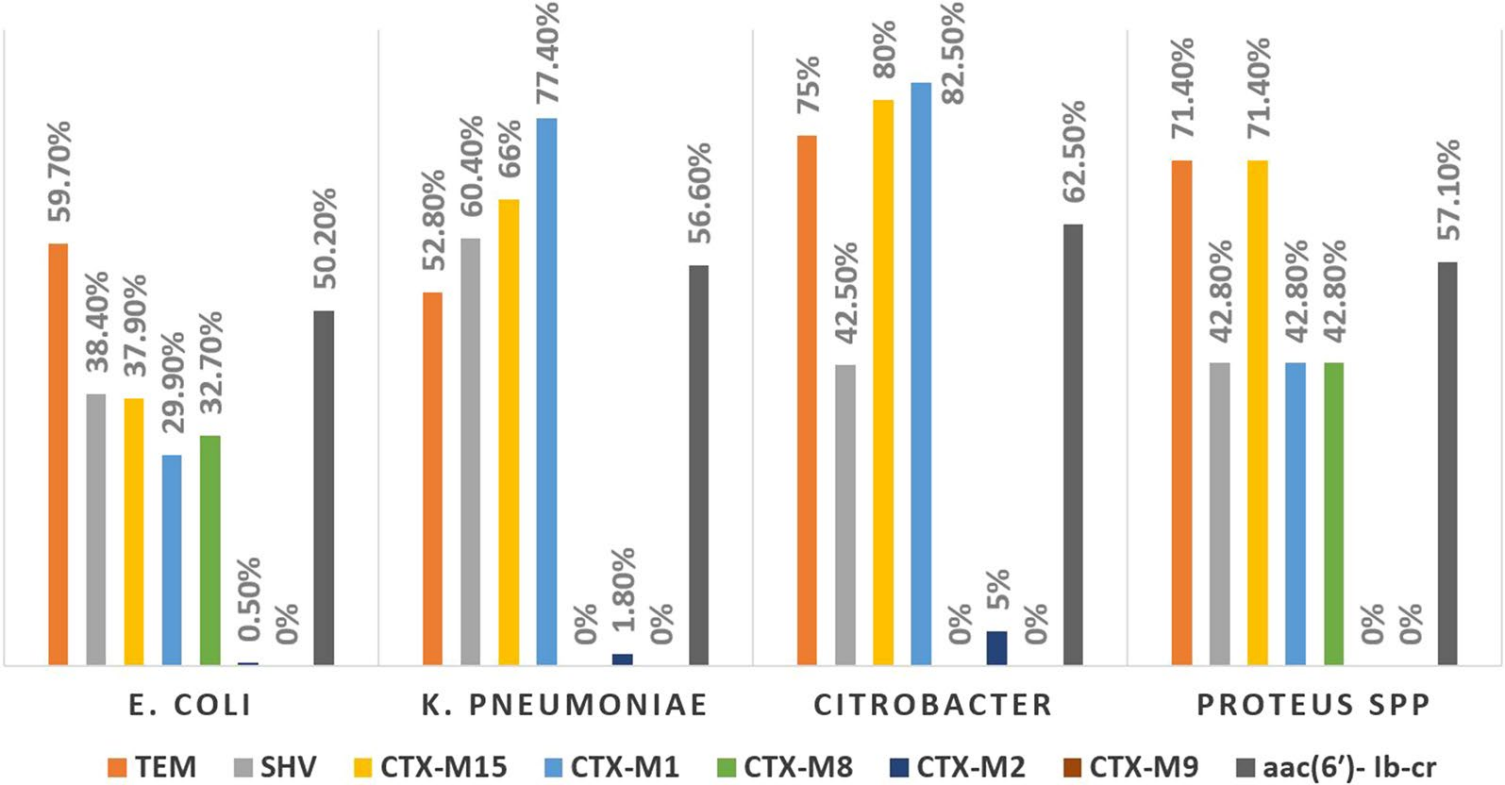
Distribution of resistance genes among 311 ESBL producing *Enterobacteriaceae* isolates

Out of 311 ESBL positive isolates, 308 (99%) isolates were positive for ESBL genes indicating high sensitivity of the phenotypic tests. *blaCTX-M* genes were detected in 254 (81.6%) isolates, out of them 19 (6.1%) harbored *blaCTX-M* alone, while the remaining 235 (75.5%) isolates harbored *blaCTX-M* in combination with *bla*TEM and/or SHV genes. However, 54 (17.3%) isolates were positive for *blaTEM* and/or *blaSHV* ESBL genes but negative for all *blaCTX-M* genes. The most prevalent gene among ESBL positive isolates was *blaTEM* gene (189, 60.7%), while within *blaCTX-M* genes, *blaCTXM-15* was the most prevalent (152, 48.8%), followed by *blaCTX-M-1* (140, 45%), *blaCTX-M-8* (72, 23.1%) and lastly *blaCTX-M-2* (4, 1.3%). The distribution of ESBL genes among different species is summarized in Fig. 2, Table 2 and (Additional file 1: Fig S1, Additional file 2: Fig S2, Additional file 3: Fig S3, Additional file 4: Fig S4). Frequency of *aac(6′)-Ib-cr* gene (responsible for resistance to AK and CIP) among ESBL producers was examined by PCR. A total of 165 (53%) isolates were positive *aac(6′)-Ib-cr* gene. The association between *aac(6′)-Ib-cr* gene and *blaCTX-M* genes was significant (*p* value < 0.01) (Table 3).

**Table 2:** Frequency and combinations of ESBL genes among phenotypically identified ESBL- producing *Enterobacteriaceae*

Genes	***E. coli*** (***n*** = 211)	***K. pneumoniae*** (***n*** = 53)	***Citrobacter*** spp. (***n*** = 40)	***Proteus*** spp. (***n*** = 7)	Total (***n*** = 311)
***blaCTX-M*** **group**					
***CTX-M-15*** **alone**	38 (18%)	0 (0%)	0 (0%)	0 (0%)	38 (12.2%)
***CTX-M-1*** **alone**	31(14.7%)	6 (11.3%)	3 (7.5%)	1 (14.3%)	41 (13.2%)
***CTX-M-1 + 15***	32 (15.2%)	35 (66%)	30 (75%)	2 (28.5%)	99 (31.8%)
***CTX-M-8*** **alone**	59 (27.9%)	0 (0%)	0 (0%)	0 (0%)	59 (19%)
***CTX-M-8 + 15***	10 (4.7%)	0 (0%)	0 (0%)	3 (42.8%)	13 (4.2%)
***CTX-M-2*** **alone**	1 (.5%)	1 (1.8%)	0 (0%)	0 (0%)	2 (0.6%)
***CTX-M-2 + 15***	0 (0%)	0 (0%)	2 (5%)	0 (0%)	2 (0.6%)
**Total**	171 (81%)	42 (79.2%)	35 (87.5%)	6 (85.7%)	254 (81.7%)
**Other β-lactamase genes**					
***blaSHV*** **only**	15(7.1%)	0(0%)	1(2.5%)	1(14.2%)	17 (5.4%)
***blaTEM*** **only**	13(6.1%)	3(5.6%)	0 (0%)	0(0%)	16(5.1%)
***blaTEM + SHV***	10(4.7%)	7(13.2%)	4(10%)	0(0%)	21(6.7%)
**Combinations**					
***blaSHV+ CTX-M***	55(26.1%)	20(37.7%)	7(17.5%)	1(14.2%)	83(26.7%)
***blaTEM*** **+** ***CTX-M***	102(48.3%)	13(24.5%)	21(52.5%)	4(57.1%)	*140(45%)*
***TEM + SHV + CTX-M***	1(.4%)	5(9.4%)	5(12.5%)	1(14.2%)	*12*(2.2%)
***CTX-M*** **genes only**	13(6.1%)	4(7.5%)	2(5%)	0(0%)	19 (6.1%)

**Table 3:** Co-carriage of ESBLs genes and *aac(6*′*)- Ib-cr* gene in *Enterobacteriaceae* isolates

***aac(6***′***)-Ib-cr*** (***n*** = 165)	Species	ESBL genes	Numbers of isolates
*aac(6*′*)- Ib-cr* associated with *CTX-M* group genes	*E. coli*	*CTX-M-15*	22
	*E. coli*	*CTX-M-15 + 1*	25
	*K. pneumoniae*	*CTX-M-15 + 1*	24
	*Citrobacter spp*	*CTX-M-15 + 1*	22
	*Proteus spp.*	*CTX-M-15 + 1*	2
	*E. coli*	*CTX-M-1*	19
	*K. pneumoniae*	*CTX-M-1*	6
	*Citrobacter spp****.***	*CTX-M-1*	3
	*E. coli*	*CTX-M-8*	2
Total			125 (75.5%)
*aac(6*′*)-Ib-cr* not associated with *CTX-M* group genes Total	*E. coli*	*SHV + TEM*	21
	*E. coli*	*SHV*	15
	*E. coli*	*TEM*	2
	*Proteus spp.*	*SHV*	2
			40 (24.2%)
*P* value			< 0.01

### Resistance pattern in ESBL genes carrying isolates and non-ESBL genes carrying isolates

The resistance rates to most of the antimicrobial agents were significantly higher in isolates carrying ESBLs genes than in isolates that don’t carry ESBL genes (p value< .05). However, the rate of resistance to cefoxitin and nitrofurantoin in the two groups did not differ significantly (p value > 0.05). (Table 4).

**Table 4:** Resistance patterns in ESBL genes carrying isolates and non-ESBL genes carrying isolates

Antibiotic	ESBL (***N*** = 308)		non- ESBL (***N*** = 132)		***P*** value
**AMC**	308	100%	43	32.6%	< 0.001
**CRO**	308	100%	35	26.5%	< 0.001
**CAZ**	308	100%	2	1.5%	< 0.0001
FOX	30	9.7%	5	3.7%	0.06
GEN	225	73%	13	9.8%	< 0.001
**AK**	90	29.2%	0	0%	0.02
**IPM**	0	0%	0	0%	–
**CIP**	90	29.2%	0	0%	0.02
**NIT**	88	28.5%	22	16.6%	0.08
**MDR**	88	28.5%	2	1.5%	0.04

*AMC* amoxicillin clavulanic acid, *CRO* ceftriaxone, *CAZ* ceftazidime, *FOX* cefoxitin, *CN* gentamicin, *AK* amikacin, *IPM* imipenem, *CIP* ciprofloxacin, *F* nitrofurantoin

### Detection of AmpC β-lactamase genes

Among 35 isolates identified as AmpC -producers by phenotypic method, 18 (51.4%) were identified as carrying AmpC genes by multiplex PCR. Among AmpC genes, DHA gene was the commonest (15, 42.3%), while FOX gene was not detected in the isolates. ESBL genes were detected in 17/18 (94.4%) of AmpC genes-carrying isolates. (Table 5).

**Table 5:** Frequency of AmpC genes among cefoxitin-resistant isolates and its combinations with ESBL genes

AmpC genes	***E. coli*** (***n*** = 18)	***K. pneumoniae*** (***n*** = 12)	***Citrobacter spp.*** (***n*** = 5)	AmpC positive (***n*** = 35)	Associated ESBL genes
MOX	0(0%)	1(8.3%)	0(0%)	1(2.8%)	*CTX-M-1 + 15*
FOX	0(0%)	0(0%)	0(0%)	0(0%)	
DHA	9 (50%)	3(25%)	0(0%)	12(34.3%)	*CTX-M-15 (6)* *CTX-M-1 (3)* *TEM (2)* No ESBL genes (1)
CIT	0(0%)	1(8.3%)	0(0%)	1(2.8%)	*CTX-M-1 + 15+ TEM*
DHA+ CIT	0(0%)	1(8.3%)	0(0%)	1(2.8%)	*CTX-M-1 + 15*
MOX + CIT	0(0%)	1(8.3%)	0(0%)	1(2.8%)	*CTX-M-1 + 15 + TEM*
MOX + CIT+ DHA	0(0%)	2(16.6%)	0(0%)	2(5.6%)	*CTX-M-1 + 15*
**Total**	9(50%)	9(75%)	0(0%)	18(51.4%)	(17/18, 94.4%)

## Discussion

Resistance of *Enterobacteriaceae* to third generation cephalosporins is a worldwide problem [27], which is mainly caused by ESBLs production. Production of additional β-lactamases (AmpC) also contributes to this problem, moreover, the presence of AmpC genes is often associated with multidrug resistance [10]. Previously, AmpC -β-lactamase has received less attention, but is now identified as an important cause of resistance in *Enterobacteriaceae* species [10]. Global spread of β*-*lactamases-producing strains gives a great importance to the study of these strains in community and hospitals for reassessment of the existing treatment protocols. In Egypt, multiple studies have investigated the prevalence of ESBLs among *Enterobacteriaceae* isolated from hospital and community acquired-UTIs [28–30]. However, little data exist on the frequency of co-existence of ESBLs and AmpC β-lactamase in different *Enterobacteriaceae* species isolated from community acquired-UTIs. The current study showed that 311/ 440 (70.6%) *Enterobacteriaceae* strains isolated from community acquired-UTIs are ESBL producers. This high frequency is comparable to a recent data reported by Hassuna et al., 2020 in our region, where 57.9% of *E. coli* isolated from community-acquired UTIs were ESBL producers [30]. On the other hand, our prevalence of ESBL-producing isolates is quite higher than that reported in several previous Egyptian studies; 17% by Fam et al., 2011 [28] and 38.8%, by Shash et al., 2019 [31], suggesting an increasing rate of ESBLs-producing *Enterobacteriaceae spread* in Egypt, that may be caused by extensive use of 3rd generation cephalosporines as empiric treatment in Egypt. The prevalence of ESBL production varies according to species, geographical areas, variations in infection control programs, different patterns of empiric antibiotic regimens and even over time*.* Moreover, selective pressure caused by the overuse of cephalosporins in some countries leads to the emergence of increasing rates of ESBLs production [32]. The prevalence of ESBL-production among species of our study was as follows; 100, 74.6, 69.6 and 46.6% of *Citrobacter* spp., *K. pneumoniae, E. coli*, and *proteus* spp*.* respectively. These findings disagree with some previous studies in Egypt, where ESBL-production was more frequent in *E. coli* isolates (17% *E. coli* and 1.2% of non-*E. coli* isolates) [28] and (97% *E. coli*, 82.6% *K. pneumoniae* and 82% *Proteus*) [33]. However, our finding was comparable with several studies from other African countries, that analyzed ESBL producing- *Enterobacteriaceae* isolated from different clinical samples. The prevalence in Uganda was 64.9% (72.7% *K. pneumoniae* and 58.1% *E. coli*) [34], in Burkina Faso was 58% (62.7% *K. pneumoniae* and 58.7% *E. coli* [35], and in Ethiopia 50.7% (52.2% *E. coli* and78.6% *K. pneumoniae*) [36]. However, our prevalence was higher than those found in USA, Europe [37], Australia [38], and also some Asian countries [39, 40]. ESBL producing *Enterobacteriaceae* isolates showed higher rates of resistance to all studied antimicrobials compared to the non-ESBL-producing isolates except for imipenem, where all tested isolates were imipenem-sensitive, that agrees with other Egyptian studies [30, 33]. On the other context, a recent study from our region reported that, (31%) of *K. pneumoniae* isolated from hospital infections were resistant to imipenem [41]. Although MDR rate among ESBL producers in the current study (28.5%) was lower than that reported in previous studies; (96.3%) [36] and (77.6%) [40], there was statistically significant increase in MDR rate reported in the ESBL-producers (28.5%) than that reported in the non-ESBL-producers (1.5%) (*p* value = 0.04). Out of 311 ESBL- producing isolates in the current study, 308 (99%) isolates were positive for ESBL genes, with *blaCTX-M* type as the most predominant. The frequency of community-acquired infections caused by *blaCTX-M*-producing strains have markedly increased in the last decade [42], that agrees with our findings, where *blaCTX-M* genes were detected in 254 (81.6%) of *Enterobacteriaceae* isolates. Within different *blaCTX-M* genes, *blaCTXM-15* was the commonest, (152, 48.8%), followed by *blaCTX-M-1* (140, 45%), then *blaCTX-M-8* (72, 23.1%). Our results concur with several studies on hospital and community-acquired infections, those reported high prevalence of *blaCTX-M* genes, particularly *blaCTX-M-15* among *Enterobacteriaceae* species in Egypt [28, 30, 33], Burkina Faso [35], Iran [38], Qatar [40] and Japan [43]. *blaTEM* and *blaSHV*-producing strains were reported previously as hospital pathogens until the late 1990s [42], however *blaTEM* and *blaSHV* gene were highly frequent among our isolates (189, 60.7%) and (133, 42.8%) respectively, this may be caused by previous contact with health care workers. This higher frequency of *blaTEM* gene in our report and also in a recent report from our region may indicate that *blaTEM* gene may be endemic in our locality [30]. Co-carriage of multiple ESBL genes in the same isolate was detected previously in Egypt [29, 30] and other countries; Burkina Faso [35], Qatar [40] and Iran [44], that concurs with our study, where 235 isolates (75.5%) harbored *blaCTX-M* in combination with *blaTEM* and/or *blaSHV* genes. AmpC β-lactamase production was identified phenotypically in 35 (7.9%) of the study isolates that was comparable with previous studies in Egypt [15, 45] and neighboring countries [46, 47]. However, another previous study in Egypt reported a higher rate (76.9%) [48]. AmpC genes were detected by multiplex PCR in 18/35 (51.4%) of cefoxitin resistant isolates, that disagrees with a previous study in Egypt that reported (88.46%) of cefoxitin resistant isolates were AmpC genes positive by PCR assay [48]. Among AmpC genes, DHA gene was the commonest (15/35, 42.3%), that disagrees with previous studies in Egypt, where CIT gene was the commonest [45, 48]. Co-carriage of AmpC genes was found exclusively in *K. pneumoniae* isolates that agrees with previous reports from Egypt and North Africa [15, 49]. Although FOX gene was commonly detected in previous Egyptian studies [15, 48], it is not detected at all in the current study. ESBL genes were detected in 17/18 (94.4%) of AmpC genes-carrying isolates, that was also reported previously [50]. The spread of ESBL genes is related to different mobile genetic elements, such as plasmid, transposons, and integrons. The co-carriage of ESBL and other-resistant genes in the same transposable genetic elements explain the co-resistance of ESBL producers to variable antibiotics. Our study investigated the frequency of *aac(6*′*)-Ib* gene among ESBL-producing *Enterobacteriaceae*, that was high rate (53%)*,* particularly among *blaCTX-M*-carrying strains (75.5%). The association between *aac(6′)-Ib-cr* gene and *blaCTX-M* genes was statistically significant (*p*-value < 0.01). This finding may explain why resistance to CIP, CN and AK was significantly higher in ESBL producers than in the non-ESBL-producers, that findings are compatible with several previous studies [39, 51, 52].

## Conclusion

Our study detected high prevalence of ESBL- production among isolated from community- acquired UTIs in south Egypt, however the prevalence AmpC β -lactamase production is low. Imipenem can be the drug of choice for community -acquired UTIs caused by these organisms*.* The *blaCTX-M* type was the predominant among ESBL-producing *Enterobacteriaceae*, especially in combination with *blaTEM* enzymes. β -lactamases production is an important cause of multiple drug resistance.

## Supplementary Information

**Additional file 1**. **Figure S1**: Agarose gel electrophoresis (2%). lane 1; molecular size marker (100 bp), lanes: 2, 4,5,8,9 are positive for *blaCTXM15* (996 bp).

**Additional file 2**. **Figure S2**: Agarose gel electrophoresis (2%). lane 1; molecular size marker (100 bp), lanes: 2, 4, 5, 6 are positive for *blaCTX-M2* (552bp).

**Additional file 3: Figure S3**. Agarose gel electrophoresis (2%). lane 1; molecular size marker (100 bp), lanes: 5, 6 are positive for *blaCTX-M8* (666bp).

**Additional file 4: Figure S4**. Agarose gel electrophoresis (2%). lane 1; molecular size marker (100 bp), lanes: 9, 10 are positive for *blaCTX-M1* (850bp), lanes: 4, 5, 6 are positive for *aac(6′)-Ib-cr* gene (482 bp).

## Data Availability

All data generated or analyzed during this study are included in this article [and its supplementary information files].

## Abbreviations

AmpC: AmpC beta lactamase
CFU: Colony-forming units
DDST: Double-disc synergy test
ESBLs: Extended-spectrum β-lactamases
MDR: Multiple drug resistance
UTIs: Urinary tract infections
