# Supplementary figures and images for: Prevalence and molecular characteristics of ESBL and AmpC β -lactamase producing Enterobacteriaceae strains isolated from UTIs in Egypt
Source: Antimicrob Resist Infect Control. 2020 Dec 10;9:198. doi: 10.1186/s13756-020-00856-w (PMC7727156; doi:10.1186/s13756-020-00856-w)

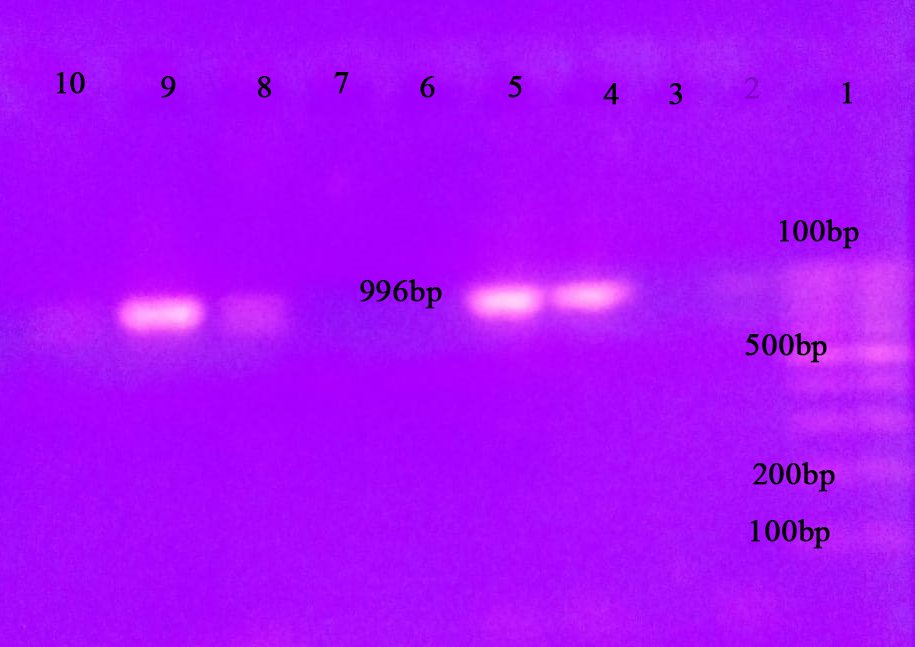

Supplement: Supplementary file 1 — Additional file 1. Figure S1: Agarose gel electrophoresis (2%). lane 1; molecular size marker (100 bp), lanes: 2, 4,5,8,9 are positive for blaCTXM15 (996 bp). [file 13756_2020_856_MOESM1_ESM.jpg]

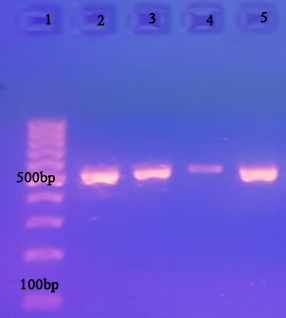

Supplement: Supplementary file 2 — Additional file 2. Figure S2: Agarose gel electrophoresis (2%). lane 1; molecular size marker (100 bp), lanes: 2, 4, 5, 6 are positive for blaCTX-M2 (552bp). [file 13756_2020_856_MOESM2_ESM.jpg]

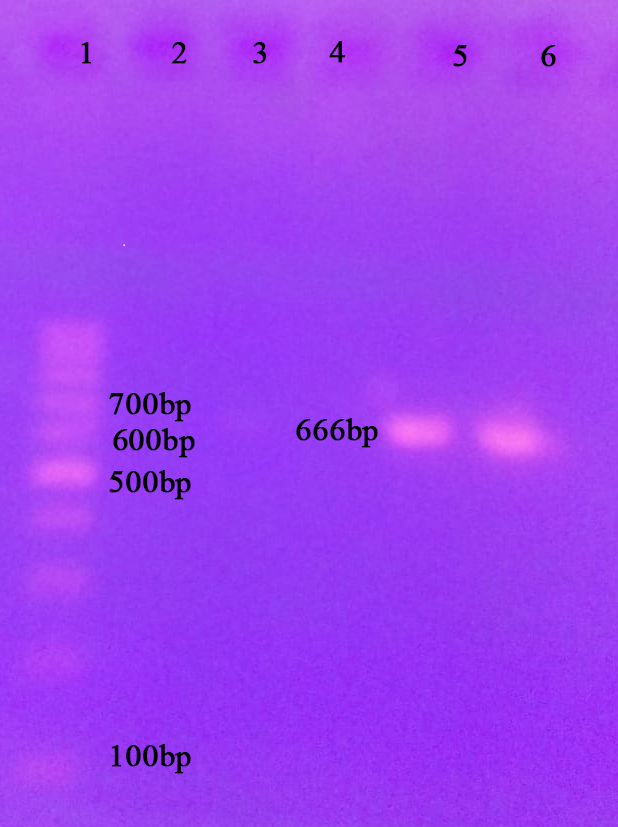

Supplement: Supplementary file 3 — Additional file 3: Figure S3. Agarose gel electrophoresis (2%). lane 1; molecular size marker (100 bp), lanes: 5, 6 are positive for blaCTX-M8 (666bp). [file 13756_2020_856_MOESM3_ESM.jpg]

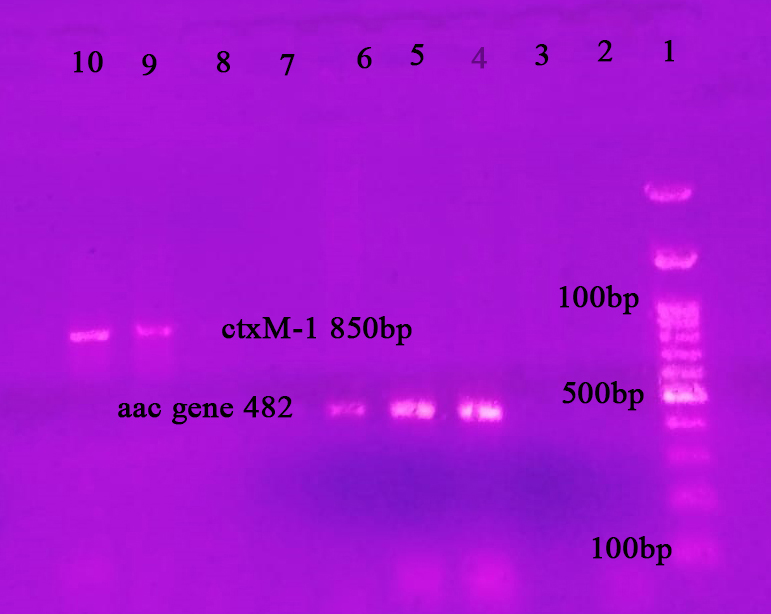

Supplement: Supplementary file 4 — Additional file 4: Figure S4. Agarose gel electrophoresis (2%). lane 1; molecular size marker (100 bp), lanes: 9, 10 are positive for blaCTX-M1 (850bp), lanes: 4, 5, 6 are positive for aac(6′)-Ib-cr gene (482 bp). [file 13756_2020_856_MOESM4_ESM.jpg]
